# The effect of exercise intention on exercise behavior in the post-epidemic era: The moderator role of openness personality and the mediated role of exercise-induced feeling

**DOI:** 10.3389/fpsyg.2022.1050918

**Published:** 2022-12-19

**Authors:** Qi-Shuai Ma, Shu-Jun Yao, Hua-Rui Jia

**Affiliations:** ^1^School of Physical Education, Huaibei Normal University, Huaibei, China; ^2^Department of Physical Education, Guang Dong Technology College, Zhaoqing, China; ^3^Department of Recreational Sports, Mokpo National University, Muan, South Korea

**Keywords:** post-epidemic era, exercise behavior, exercise intention, openness personality, exercise-induced feeling

## Abstract

**Objective:**

Based on the theory of planned behavior, this study introduced personality traits and exercise-induced feelings and other third variables, aiming to explore the mechanism of personality traits and exercise-induced feelings between exercise intention and exercise behavior, and explore the internal mechanism of promoting exercise behavior of junior high school students.

**Methods:**

This research adopts the Exercise Intention Questionnaire, Simple Big Five Personality Scale, Exercise-induced Feeling Inventory and Exercise Rating Scale, from the three cities of Anhui province 1,166 junior high school students selected from the group psychological measurement, and uses the SPSS23.0 and Process plug-in exercise intention in exercise behavior analysis of the direct and indirect effect.

**Results:**

The results showed that: (1) Exercise intention significantly positively predicted exercise behavior (*β* = 0.265, *t* = 4.261, *p* < 0.01). (2) The moderating effect of openness personality between exercise intention and exercise behavior was significant (*β* = 0.093, *t* = 4.431, *p* < 0.01). (3) Exercise-induced feelings has a significant mediating effect on the relationship between exercise intention and exercise behavior regulated by openness personality.

**Conclusion:**

Exercise intention can effectively predict exercise behavior, and the prediction level is affected by openness personality, and the moderating effect of openness personality is partially realized through the mediating variable of exercise-induced feelings.

## Introduction

The COVID-19, a public health emergency, has greatly affected the behavior of Chinese junior high school students. Students stay indoors, and the courses are changed to “online” teaching, especially physical education, which can not meet the exercise needs of junior high school students and greatly affect their physical and mental health. With the arrival of the post epidemic era, the school has organized students to return to school on a large scale, face-to-face classes have gradually returned to the normal track, and physical education has also returned to the playground. However, the loss of physical and mental health caused to junior high school students during the epidemic is difficult to recover. Therefore, how to improve the exercise behavior of students in the post epidemic era has become a hot topic in exercise psychology (Huang et al., 2021).

The social anxiety brought by the epidemic has greatly affected the mental health of junior high school students. Physical exercise is recognized as the most effective way to “reduce stress,” and a large number of studies have confirmed that moderate physical exercise can help improve the cognitive ability of junior high school students (Wen, 2015). Improve their academic performance (Grigolava et al., 2021), improve their interpersonal skills (Chen et al., 2020; Montse and Claudio, 2020), cultivate their self-esteem and self-confidence (Pan et al., 2022), and promote the development of their social adaptability (Wan et al., 2021). Therefore, it is an important significance to explore the relationship between exercise behavior and its influencing factors. Nowadays, the research on the influencing factors of exercise behavior has gradually moved toward theorization and modeling. There are many psychological theories applied to the study of exercise behavior, such as self-determination theory, expected value theory, stage development theory and planned behavior theory, of which the theory of planned behavior (TPB) is the most widely used. A large number of studies have explored the intention-behavior relationship in adolescents on the basis of the TPB, but current research cannot adequately explain the mechanisms by which intention-behavior occurs and it is unclear how to bridge the gap between intention and behavior (Xiang, 2016), in addition to which few studies have been conducted on the exercise behavior of secondary school students in the post-epidemic period. Based on the TPB model proposed by Ajzen (2011), this paper explores the relationship between exercise behavior and its influencing factors, and provides empirical evidence for improving exercise behavior of junior high school students in the “post epidemic period.”

### Exercise intention and exercise behavior

Exercise behavior refers to sports activities aimed at developing body, improving health, strengthening physique, regulating spirit, enriching cultural life and controlling leisure time. The theory of planned behavior is one of the most important theoretical models in the field of sports (Hohmann and Garza, 2022). The theory of planned behavior holds that human behavior is the result of deliberate planning. Therefore, exercise intention can predict the generation of exercise behavior, and a large number of studies have confirmed that exercise intention is an effective predictor of exercise behavior (Chen et al., 2022; Teixeira et al., 2022; Zhang et al., 2022). Hou et al. (2022) confirmed in a study of 242 college students in China that exercise intention can effectively predict exercise behavior. Gu et al. (2022) found that exercise attitude was a significant predictor of exercise behavior based on TPA during COVID-19. Liu (2022) used meta-analysis to find that exercise intention influenced exercise behavior to some extent, but many studies have exaggerated the effect of exercise intention in predicting exercise behavior. Therefore, we propose the following hypotheses:

*H1:* Exercise intention of junior high school students can positively predict exercise behavior.

At present, relevant scholars who study the theory of planned behavior believe that the third variable often affects exercise intention in the process of affecting exercise behavior, that is, under different conditions, the degree of exercise intention affecting exercise behavior will change (Liu, 2019). In order to better promote the formation of physical exercise behavior of junior high school students, it has important research value to study how exercise intention affects exercise behavior.

### The moderator role of personality

Junior high school is the golden age of individual development, and it will also face various great challenges. With the arrival of adolescence, there will be rapid changes both physically and psychologically (Yang and Ma, 2014; Heilmann et al., 2021). Personality is one of the important factors that affect how individuals adapt to these changes after entering junior high school. Since Goldberg (1990) discovered the five personality factors through factor analysis, psychologists have invested a lot of energy in exploring the five personality factors (Ka et al., 2021). It is found that having different personalities will have a significant impact on individual psychological and behavioral development (Wilks et al., 2020; Pilch et al., 2021). Van den Akker et al. (2021) found that as adolescents enter puberty, their levels of responsibility, extraversion and openness personality traits will temporarily decline, and their neuroticism personality levels will increase. In fact, this represents a decline in individual personality maturity. At the same time, a study showed that junior high school students’ exercise behavior was significantly reduced in adolescence (Cao et al., 2011). So, will personality have a significant impact on exercise behavior? Bandura’s (2004) theory of interactive personality in behaviorism holds that behavior is the result of the combination of personality traits and situations, and situations have strong control over behavior. When predicting behavior, the focus should be on the specific stimulus conditions that can be observed, not on the intrinsic characteristics. Individuals are the active constructors of situations, and situations are the results of interaction between social individuals and the objective environment. That is to say, behavior will be affected by the interaction between individuals and situations. Behavior is often the result of the interaction between individual cognition and the situation, and any individual has its distinctive personality traits. Therefore, under different stimulus conditions (personality traits), the execution of intention will be strengthened or weakened. Research shows that during the COVID-19 epidemic, long-term closure of schools will have harmful mental health consequences for teenagers (Kim et al., 2021). And individuals with higher openness personality have a higher degree of deterioration of mental health (Proto and Zhang, 2021). The characteristic of openness personality is imaginative creative and curious, and closed management during the epidemic may have a greater impact on individuals with openness personality. Research shows that a high level of openness personality and extraversion personality will be more conducive to individuals to put into action and enhance their execution of exercise intentions (Han et al., 2021). And the low-level openness personality does not care about his own ideas and is unwilling to take action (Nave et al., 2016). Therefore, we speculate that personality may affect the extent to which an individual’s intention predicts behavior. Based on this, we propose the following hypotheses:

*H2:* Personality plays a moderating role in the relationship between exercise intention and exercise behavior of junior high school students.

### The mediated role of exercise-induced feeling

Feeling usually refers to people’s attitude toward and experience of objective things and corresponding behavioral reactions, and is considered as a psychological activity when individuals carry out activities (Heintzelman and King, 2014). Previous studies on exercise intention and exercise behavior mainly focused on their relationship, but few focused on the specific mechanism of the effect of exercise intention on junior high school students’ exercise behavior (Mao and Guo, 2013). According to the triadic reciprocal determinism (Bandura, 1989), behavior will be affected by the interaction between environment, thinking, cognition and other individual subjective factors. Research has confirmed that exercise intention will be affected by the individual’s surroundings (Guo et al., 2022). The difference in emotional benefits between individuals mainly comes from the difference in cognition between individuals, that is, behavior may be affected by both intention and feeling benefits (Liu and Wang, 2020). Research shows that low level of individual intention will inhibit the generation of positive emotions, and bad emotional experience will reduce the enthusiasm of individuals to engage in activities and discourage enthusiasm (Zhang et al., 2014). A high level of exercise intention will significantly affect the development of exercise feelings. The stronger the intention is, the easier it is for individuals to experience positive feelings (Zhang and Mao, 2016). In addition, the high level of feeling benefits of exercise is also the source of promoting individual exercise behavior (Huellemann et al., 2021). Because the exercise-induced feeling is the result of individuals’ high recognition of exercise intentions, it has become the most direct motivation for junior high school students to actively participate in exercise activities, and can help them develop good exercise habits. At the same time, junior high school students who have bad feeling benefits of exercise are prone to the phenomenon of inactivity. It can be inferred that while improving the level of exercise intention of junior high school students, it may also improve their cognition of physical exercise, meet their own exercise needs and interpersonal needs, promote their participation in exercise activities to bring more feeling benefits, and thus affect the behavioral results. Thus, we propose the following hypotheses:

*H3:* Exercise-induced feeling plays a mediated role in the relationship between exercise intention and exercise behavior of junior high school students.

Does the mediating effect of exercise-induced feeling still exist in the path of personality traits and exercise behavior? Feeling is not only the core factor of personality formation, but also related to all aspects of social life, playing a pivotal psychological role in coordinating various elements of social life (Damasio, 2021). According to embodied emotion theory, feeling and body change are unified. In the process of emotional information processing, they are an interactive whole. The generation of feeling is the result of the interaction between body, mind and the external world (Burkitt, 2021). Under the framework of embodied cognition, cognition and feeling work together, making it easier for human beings to adapt to the challenge of evolution (Xu and Zhang, 2021). Good ability to regulate feeling can promote the development of physical and mental health, while cognition can promote individual feeling regulation, and exercise intention comes from individual cognition of their own needs (Hermann et al., 2021). Regular physical exercise can promote the individual’s ability to adjust feelings (Perchtold-Stefan et al., 2020). Previous studies have found that exercise-induced feeling has a significant positive predictive effect on personality and motivation (Zhu and Dong, 2016). Self-determination theory believes that personality traits have an important impact on emotion or emotion regulation strategies (Sheldon and Prentice, 2019). Exercise-induced feeling is a cognitive factor, and the change of psychological environment can significantly affect emotion. Halevy et al. (2019) comprehensively investigated the relationship between feeling, psychological environment factors and behavior results, and found that feeling can affect behavior results by influencing psychological environment. Kim (2022) found that the openness personality and extraversion personality in personality traits can improve junior high school students’ motivation during exercise, meet the needs of students’ autonomy, interpersonal relationship and ability development, and thus enhance their exercise induced emotions. Thus, we propose the following hypotheses:

*H4:* The moderating effect of personality traits on exercise intention and exercise behavior may be realized through the mediating effect of exercise-induced feeling.

**Figure 1:**
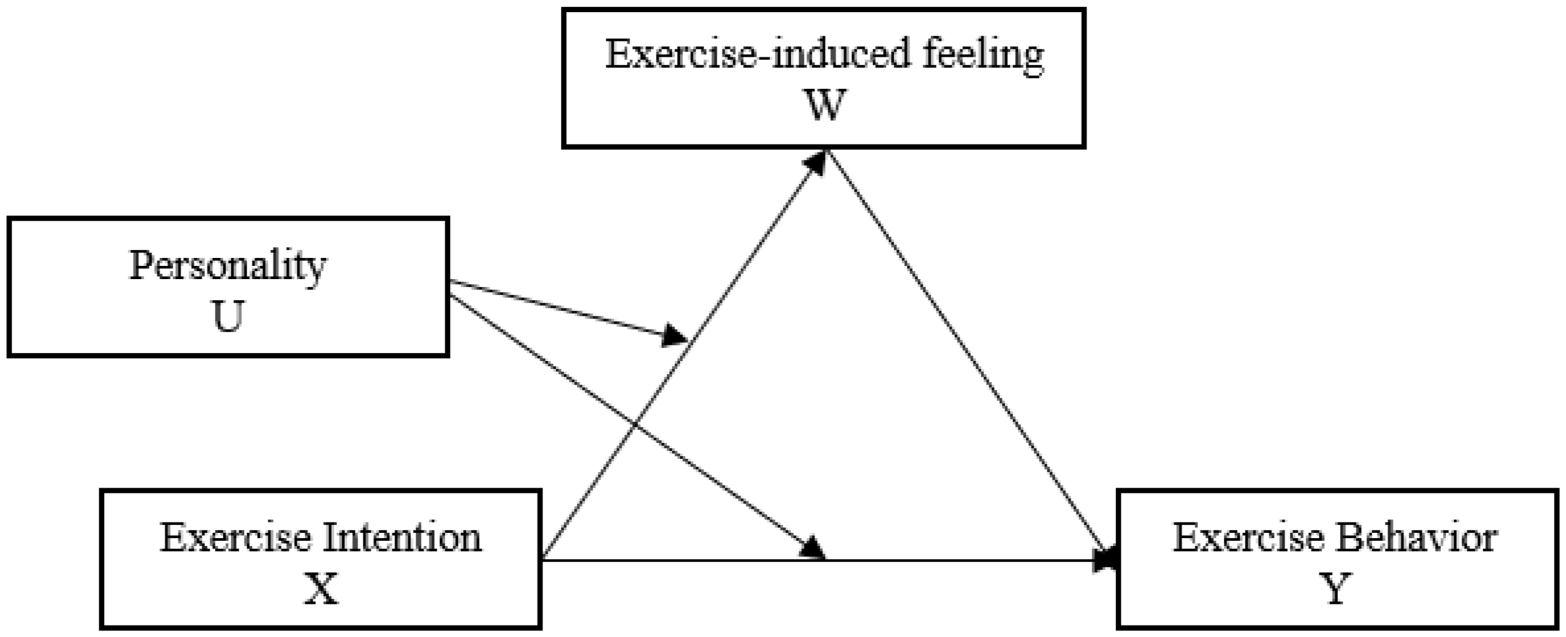
Hypothetical model.

To sum up, this study built a conceptual model of exercise intention and exercise impact mechanism (Figure 1).

## Materials and methods

### Procedure and participants

From September to October 2021, a cross-sectional survey was conducted by using the convenience sampling method in Anhui Province. This study selected one junior high school in urban and rural areas in the north, middle and south of Anhui Province (6 junior high schools in total). Two classes will be randomly selected from each grade of each junior high school (36 classes in total), and 1,355 questionnaires will be issued. The students were tested in the classroom, and the main testers were all psychology students who had received professional training. The test was approved by the school leaders, head teachers and participants, and all questionnaire were completed within 10 min. After the questionnaire was collected, the invalid questionnaires caused by regular answers and missing data were removed, and 1,166 valid questionnaires were recovered, with a recovery rate of 86.05%. The participants age ranging from 11 to 17 years (*M*_age_ = 14.51, SD_age_ = 0.66), including 581 boys and 585 girls. There are 391 students in Grade One, 389 students in Grade Two and 386 students in Grade Three.

The study complied with the principles of the Declaration of Helsinki, and it is supported and approved by the Institutional Review Board of Huaibei Normal University. Parents or guardians of all participants signed informed consent. The informed consent described the purpose and process of the study, the method used and publication plans. It also included confidentiality assurance, the principles for voluntary participation and included contact information to the researcher and organization behind the study.

### Demographic characteristics of the study sample

As shown in Table 1, of the total sample, 49.83% (581) were boys, and 50.17% (585) were girls. The exercise intention level of boys was significantly higher than that of girls, personality of extraversion level of boys was significantly lower than that of girls, personality of conscientiousness level of boys was significantly higher than that of girls, exercise behavior level of boys was significantly higher than that of girls, and there was no significant difference between boys and girls in exercise-induced feeling, agreeableness, neuroticism and openness.

**Table 1:** Differences in gender.

Variable	Gender	Number (%)	*M*	SD	*t*	*p*
EI	Male	581(49.83)	6.32	1.25	7.372	<0.001
	Female	585(50.17)	5.79	1.23		
EIF	Male	581(49.83)	3.91	0.43	1.475	0.119
	Female	585(50.17)	3.82	0.40		
Extraversion	Male	581(49.83)	2.77	0.25	−2.394	0.038
	Female	585(50.17)	2.89	0.27		
Agreeableness	Male	581(49.83)	3.29	0.51	1.576	0.095
	Female	585(50.17)	3.27	0.43		
Conscientiousness	Male	581(49.83)	3.59	0.44	5.469	<0.001
	Female	585(50.17)	3.17	0.39		
Neuroticism	Male	581(49.83)	3.53	0.57	0.713	0.261
	Female	585(50.17)	3.52	0.51		
Openness	Male	581(49.83)	3.61	0.42	0.841	0.219
	Female	585(50.17)	3.57	0.61		
EB	Male	581(49.83)	22.71	7.54	8.816	<0.001
	Female	585(50.17)	18.74	6.55		

EI, exercise intention; EIF, exercise-induced feeling; EB, exercise behavior.

As shown in Table 2, of the total sample, 33.53% (391) were grade one students, 33.36% (389) were grade two students, and 33.10% (386) were grade three students. There are no significant differences in each variable among different grades.

**Table 2:** Differences in grade.

Variable	Grade	Number (%)	*M*	SD	*F*	*p*
EI	One	391(33.53)	5.81	1.23	2.46	0.659
	Two	389(33.36)	5.88	1.22		
	Three	386(33.10)	5.97	1.20		
EIF	One	391(33.53)	3.88	0.57	1.48	0.753
	Two	389(33.36)	3.84	0.52		
	Three	386(33.10)	3.86	0.55		
Extraversion	One	391(33.53)	2.84	0.23	1.87	0.712
	Two	389(33.36)	2.86	0.21		
	Three	386(33.10)	2.82	0.21		
Agreeableness	One	391(33.53)	3.31	0.57	2.35	0.675
	Two	389(33.36)	3.25	0.55		
	Three	386(33.10)	3.27	0.54		
Conscientiousness	One	391(33.53)	3.49	0.63	3.94	0.521
	Two	389(33.36)	3.37	0.68		
	Three	386(33.10)	3.34	0.66		
Neuroticism	One	391(33.53)	3.48	0.59	2.73	0.618
	Two	389(33.36)	3.55	0.61		
	Three	386(33.10)	3.38	0.64		
Openness	One	391(33.53)	3.68	0.66	1.98	0.703
	Two	389(33.36)	3.49	0.58		
	Three	386(33.10)	3.55	0.60		
	One	391(33.53)	18.76	7.49		
EB	Two	389(33.36)	20.74	7.51	2.51	0.634
	Three	386(33.10)	22.23	6.58		

EI, exercise intention; EIF, exercise-induced feeling; EB, exercise behavior.

### Measures and instruments

#### Exercise intention

*Exercise Intention Scale* was compiled by Ajzen (2006). Fang (2012) translated and revised this scale to measure the exercise intention among Chinese junior high school students. This study adopted revised *Exercise Intention Scale* by Fang to measure exercise intention. It is a 7-point-Likert scale consisting of 3 items. (e.g., “I plan to exercise for at least 20 min at least three times a week, in the next 2 weeks,” “I intend to exercise for at least 20 min at least three times a week, in the next 2 weeks” and “I hope to exercise for at least 20 min at least three times a week, in the next 2 weeks.”). Each item is rated from 1 (completely disagree) to 7 (completely agree), use average score to represent individual exercise intention and a higher score means a stronger the intention of exercise. Previous research has confirmed that *Exercise Intention Scale* has good reliability and validity among Chinese junior high school students (Liu, 2022). In the present study, confirmatory factor analysis results demonstrated that a single-factors model fit the data satisfactorily: *χ^2^/df* = 4.19, CFI = 0.91, TLI = 0.89, RMSEA = 0.049, SRMR = 0.048, and the Cronbach’s *α* was 0.86.

#### Personality

*NEO-PI-R (Revised NEO Personality Inventory) Scale* was compiled by Costa and McCrae (1995) and it is the most widely used tool for measuring the five-factor model of personality (Jiang et al., 2022). Wang et al. (2011) translated and revised this scale to measure the five-factor model of personality among Chinese junior high school students. This study adopted revised *Chinese Big Five Personality Inventory Scale* (CBF-PI) by Wang et al. to measure five-factor model of personality. The *CBF-PI* is a 5-point-Likert scale consisting of 10 items and 5 dimensions: extraversion (e.g., “I see myself as someone who is outgoing, sociable”), agreeableness (e.g., “I see myself as someone who is generally trusting”), conscientiousness (e.g., “I see myself as someone who does a thorough job”), neuroticism, (e.g., “I see myself as someone who gets nervous easily”) and openness (e.g., “I see myself as someone who has an active imagination”). Each item is rated from 1 (disagree strongly) to 5 (agree strongly), use average score to represent the degree of various personality traits and a higher score shows a higher level of this personality trait (Beatrice and Oliver, 2007). Previous research has confirmed that *CBF-PI* has good reliability and validity among Chinese junior high school students (Jiang et al., 2015). In the present study, confirmatory factor analysis results demonstrated that a five-factors model fit the data satisfactorily: *χ^2^/df* = 3.21, CFI = 0.97, TLI = 0.95, RMSEA = 0.052, SRMR = 0.038. The Cronbach’s *α* of the extraversion dimension was 0.91, agreeableness was 0.85, conscientiousness was 0.88, neuroticism was 0.86 and openness was 0.82.

#### Exercise-induced feeling

The *Exercise-induced Feeling Inventory* (EFI) was compiled by Gauvin and Rejeski (1993). Chen et al. (2007) translated and revised this scale to measure the exercise-induced feeling of Chinese junior high school students. This study adopted revised *EFI* by Chen et al. to measure exercise-induced feeling. The *EFI* is a 6-point-Likert scale consisting of 12 items and 4 dimensions: energize (e.g., “During or after physical exercise, I feel exhilarated”), calm (e.g., “During or after physical exercise, I feel relaxed”), exhausted (e.g., “During or after physical exercise, I feel tired”) and active, (e.g., “During or after physical exercise, I feel passionate”). Each item is rated from 1 (completely disagree) to 6 (completely agree), use average score to represent the degree of various exercise feeling and a higher score shows a higher level of conformity of this dimension (Wang, 2015). Previous research has confirmed that *EFI* has good reliability and validity among Chinese junior high school students (Liu, 2016). In the present study, confirmatory factor analysis results demonstrated that a single-factors model fit the data satisfactorily: *χ^2^/*df = 4.15, CFI = 0.88, TLI = 0.94, RMSEA = 0.051, SRMR = 0.047, The Cronbach’s *α* of the energize dimension was 0.90, calm was 0.86, exhausted was 0.91 and active was 0.89.

#### Exercise behavior

*Physical Activity Rating Scale (PARS-3)* was compiled by Liang (1994). *PARS-3* is widely used to measure the physical exercise behavior among Chinese junior high school students (Wang et al., 2022), and this study adopted it to measure exercise behavior. The *PARS-3* includs three aspects: exercise intensity (e.g., “What is the intensity of your physical exercise?”), exercise time (e.g., “How many minutes do you spend on the physical activities?”) and exercise frequency (e.g., “How many times do you do physical activities in a month?”). Each item is rated from 1 to 5, and the following equation computes the total score of physical activity: exercise intensity × (exercise time-1) × exercise frequency, with a range of 0 to 100. A higher score shows a higher amount of physical exercise. Previous research has confirmed that *PARS-3* has good reliability and validity among Chinese junior high school students (Dong and Mao, 2021). In the present study, the scale has high reliability and validity, its retest reliability *r* = 0.87, and Cronbach’s *α* was 0.89.

#### Statistical analyzes

After the questionnaire data were collected, Amos 24.0 was used for confirmatory factor analysis to test the validity of variables, and IBM SPSS23.0 for *Pearson* correlation analysis and common method bias test. According to the study of Ye and Wen (2013). Using process macro in IBM SPSS23.0 to examine our model. Firstly, we used process Model 1 to test the moderating effect of personality on exercise intention and exercise behavior. Secondly, used process Model 1 to test the moderating effect of personality on exercise intention and exercise-induced feeling. Finally, used process Model 8 to test the relationship between the moderating effect of personality on exercise intention and exercise behavior with exercise-induced feeling. During the model test, grade and gender were included in the model as covariates. The continuous variables of normal distribution were expressed as mean (M) ± standard deviation (SD) and the incomplete questionnaire has been deleted when enter data, so there is no missing date in this study. Goodness of fit index *χ*^2^/df less than 5, RMSEA less than 0.08, NNFI and CFI greater than 0.8, and SRMR less than 0.05 are acceptable. In this study, significance level was set as *p* < 0.05 and effect sizes of the correlation coefficient r (Wangzhou et al., 2021) to estimate the magnitude of significant differences during statistical analysis.

## Results

### Common method bias test

In this study, two common method bias test methods will be used to test data to ensure that there is no serious man-made covariant problem between predictive variables and standard variables (Tang and Wen, 2020). First, Harman single factor was used to test the common method bias (Aguirre-Urreta and Hu, 2019). The results showed that the characteristic roots of five factors were greater than 1, and the first factor could explain 31.032%, which was less than the standard critical value of 40%. The common method bias of this study was acceptable. Secondly, a single common method factor control method is used to test the common method bias. The results show that the model containing common method factors cannot fit the data. To sum up, the results of both test methods show that there is no obvious problem of common method bias in this study.

### Descriptive statistics and correlation analysis

Descriptive statistics and related analysis results of each research variable are shown in Table 3. Table 3 shows that exercise intention, openness personality, exercise-induced feeling, extraversion personality, conscientiousness personality, neuroticism personality are significantly positively correlated with exercise behavior of junior high school students; Agreeableness personality is negatively correlated with exercise behavior; Exercise intention, openness personality, extroversion personality, agreeableness personality, conscientiousness personality, neuroticism personality and exercise-induced feeling were significantly positively correlated; Openness personality, agreeableness personality, conscientiousness personality, neuroticism personality and exercise intention were significantly positively correlated. The relationship between variables supports subsequent hypothesis testing.

**Table 3:** Means, standard deviations, and correlations among variables.

Variable	*M*	SD	1	2	3	4	5	6	7	8
1. EI	5.93	1.22	1							
2. EB	20.91	7.78	0.265**	1						
3.Openness	3.59	0.81	0.246**	0.206**	1					
4. EIF	3.87	0.80	0.401**	0.220**	0.218**	1				
5. Extraversion	2.84	0.69	0.054*	0.074**	0.121**	0.140**	1			
6. Agreeableness	3.28	0.69	0.148**	−0.019	−0.125**	0.121**	−0.015	1		
7. Conscientiousness	3.39	0.71	0.167**	0.124**	0.234**	0.126**	−0.016	−0.037	1	
8. Neuroticism	3.52	0.82	0.310**	0.159**	0.296**	0.293**	0.123**	0.308**	0.118**	1

EI, exercise intention; EIF, exercise-induced feeling; EB, exercise behavior. ^**^p < 0.01, ^*^p < 0.5.

### Test of mediated moderating model

In this study, the five dimensions of personality of the Big Five Personality Scale were tested by regulatory analysis. The test results showed that there was no regulatory effect on extraversion personality in exercise intention and exercise behavior (*p* = 0.195 > 0.05), no regulatory effect on agreeableness personality in exercise intention and exercise behavior (*p* = 0.062 > 0.05), and no regulatory effect on conscientiousness personality in exercise intention and exercise behavior (*p* = 0.736 > 0.05) Neurotic personality has no regulatory effect on exercise intention and exercise behavior (*p* = 0.658 > 0.05), but only openness personality has significant regulatory effect on exercise intention and exercise behavior (*p* < 0.001, *t* = 4.431). Therefore, according to a procedure proposed of test mediated moderating effects by Ye and Wen (2013), this study is conducted according to the following steps:

1. Test whether the moderating effect of open personality on the relationship between exercise intention and exercise behavior of junior high school students is significant. If so, continue with the following steps. Otherwise, stop the analysis.
2. Test whether the moderating effect of openness personality on the relationship between exercise intention and exercise-induced feeling of junior high school students is significant, and whether the relationship between exercise-induced feeling and exercise behavior is significant. If both are significant, then at least one part of the moderating effect of openness personality on exercise intention and exercise behavior is realized through the intermediary variable exercise-induced feeling.
3. The interaction between exercise intention, openness personality, exercise-induced feeling and exercise intention and openness personality (U × X). The regression test of the relationship between exercise intention and exercise behavior is significant. If it is not significant, it indicates that the regulatory effect is completely mediated; If significant, it indicates that the moderating effect is partially mediated. The inspection is completed.

**Figure 2:**
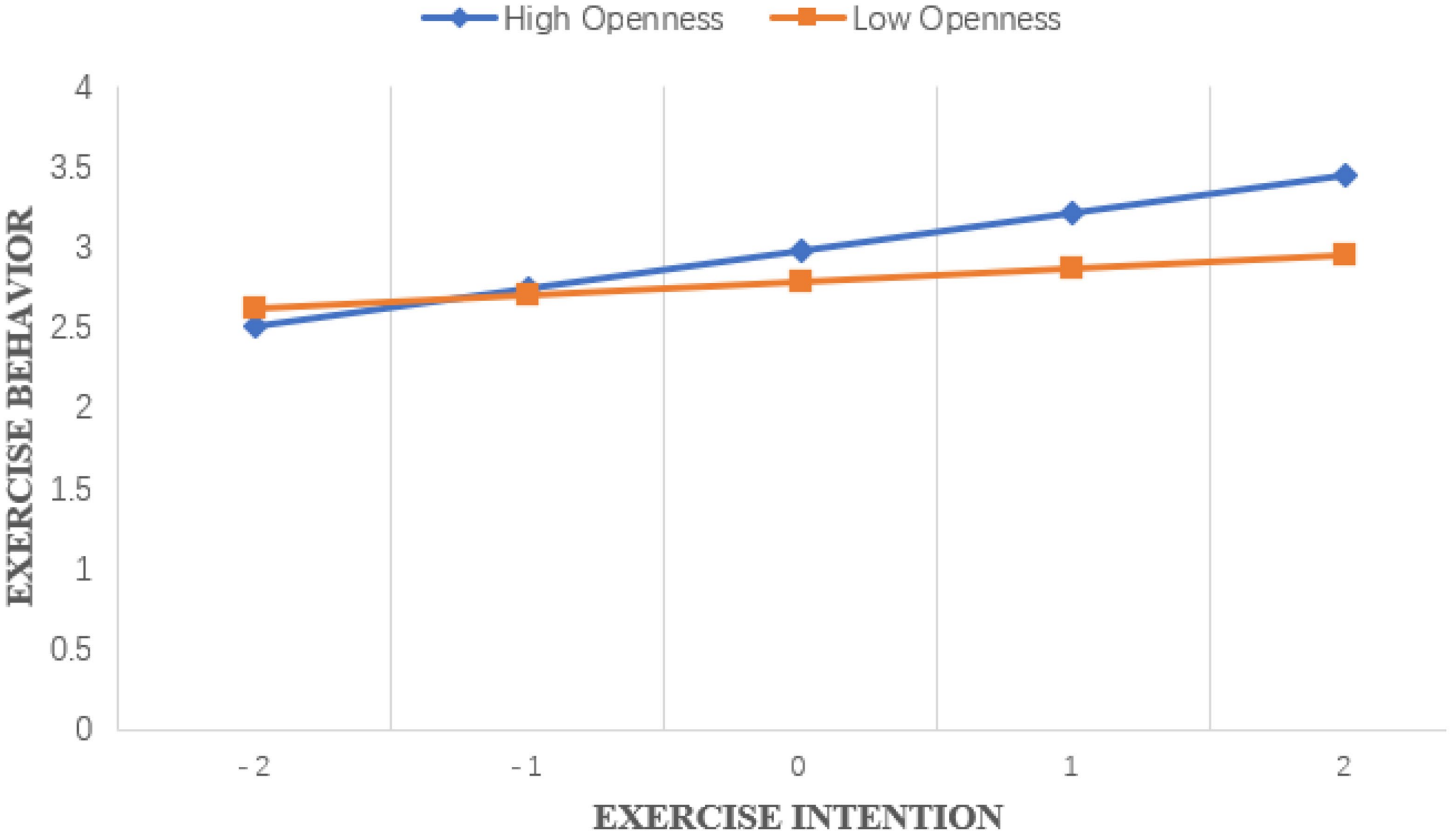
The moderating effect of openness on exercise behavior and exercise intention.

**Figure 3:**
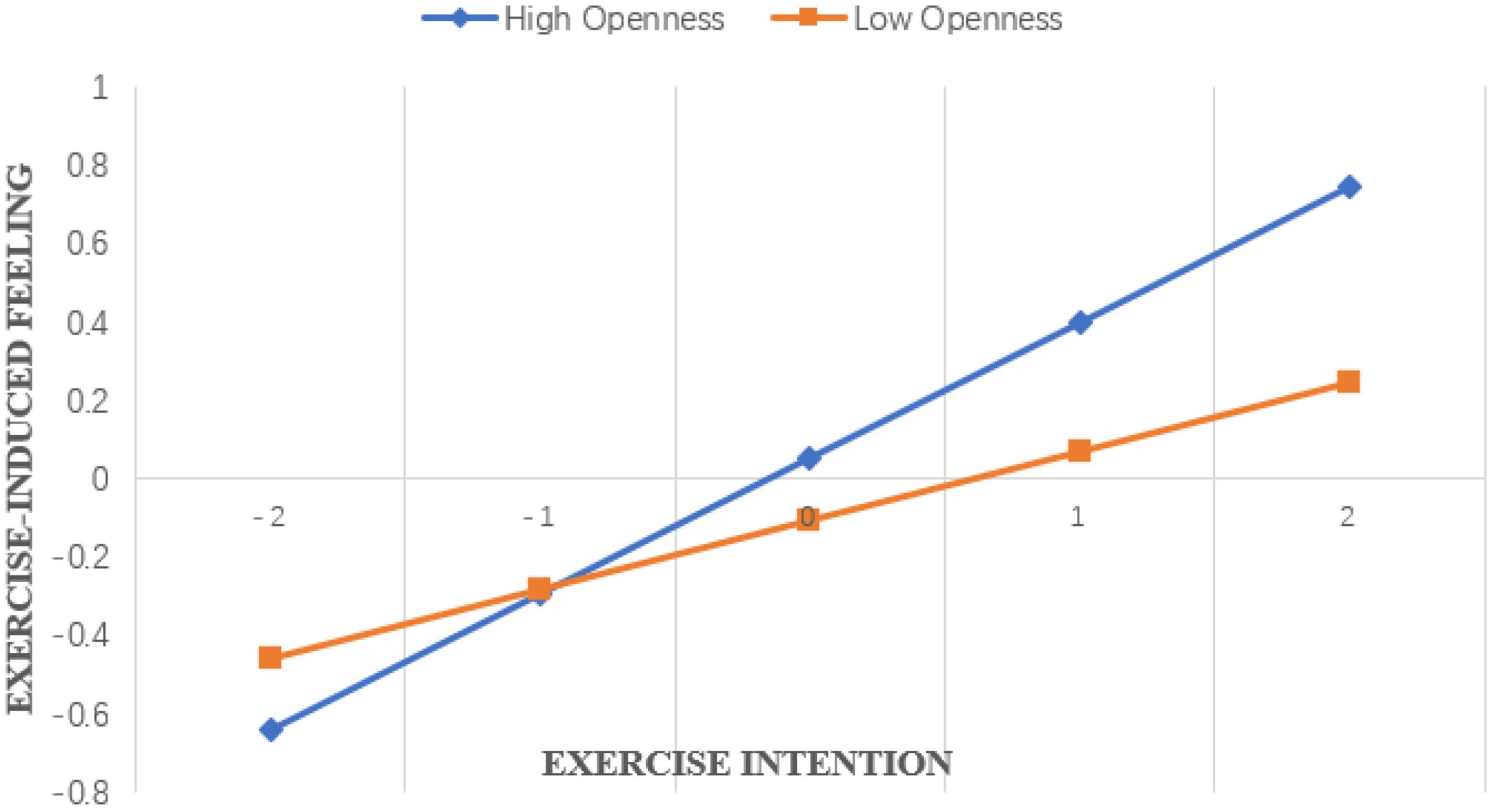
The moderating effect of openness on exercise-induced feeling and exercise intention.

**Figure 4:**
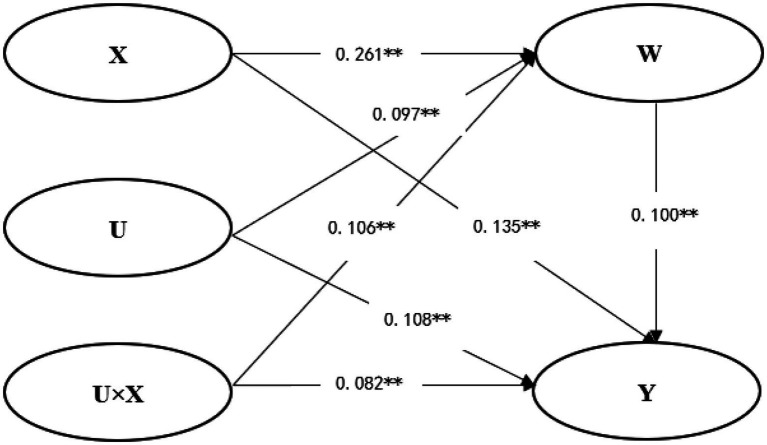
The mediated moderating model (**p <0.01).

Except for the dependent variable exercise behavior, all variables were standardized in this study. See Table 4 for specific estimated parameters. The results showed that in Table 4, junior high school students’ exercise intention, openness personality and their interaction items were significantly positive predictors of exercise behavior. Further simple slope test analysis results show that (Figure 2), when the level of openness personality is high, junior high school students’ exercise intention has a strong predictive effect on exercise behavior (*β* = 0.236, *t* = 9.175, *p* < 0.01). When the level of openness personality is low, junior high school students’ exercise intention has a weak predictive effect on exercise behavior (*β* = 0.085, *t* = 4.094, *p* < 0.01), that is, openness personality can regulate the relationship between exercise intention and exercise behavior of junior high school students. Table 4 shows that junior high school students’ exercise intention, openness personality and the interaction between exercise intention and openness personality can significantly predict junior high school students’ exercise-induced feeling. That is to say, openness personality only has a moderating effect between exercise intention and exercise-induced feeling of junior high school students. Further simple slope test shows that (Figure 3), when the level of openness personality is high, exercise intention has a strong predictive effect on exercise-induced feeling (*β* = 0.347, *t* = 13.730, *p* < 0.01). When the level of openness personality is low, the predictive effect of exercise intention on exercise-induced feeling of junior high school students is weak (*β* = 0.175, *t* = 8.540, *p* < 0.01). Table 4 and Figure 4 show that exercise-induced feeling has a significant positive predictive effect on exercise behavior, and the interaction term of exercise intention and openness personality has a significant predictive effect on exercise behavior, indicating that exercise-induced feeling of junior high school students plays a partial intermediary role in the relationship between openness personality moderated exercise intention and exercise behavior, and the intermediary effect value is 0.106 × 0.10 = 0.0106, the proportion of intermediary effect in the total effect is 0.0106/(0.0106 + 0.082) = 11.45%. To sum up, the four relationship models of junior high school students’ exercise intention, openness personality, exercise-induced feeling and exercise behavior conform to the mediated moderating model.

**Table 4:** Test of mediated moderating model.

Variable	Model 1(Output variable:EB)			Model 2(Output variable:EIF)			Model 3(Output variable:EB)		
	*β*	SE	*t*	*β*	SE	*t*	*β*	SE	*t*
Constant	2.88	0.02	148.55**	3.84	0.02	201.78**	2.89	0.02	146.15**
EI(*X*)	0.16	0.02	9.97**	0.26	0.07	16.48**	0.14	0.02	7.38**
Openness(*U*)	0.12	0.02	4.84**	0.1	0.02	4.06**	0.11	0.03	4.22**
*X* × *U*	0.09	0.02	4.43**	0.11	0.02	5.15**	0.08	0.02	3.60**
EIF(W)							0.1	0.03	3.66**
*R* ^2^	0.102			0.189			0.111		
Δ*R*^2^	0.101			0.188			0.108		
*F*	*F*(3,1,569) = 59.695, *p* = 0.000			*F*(3,1,569) = 122.211, *p* = 0.000			*F*(4,1,568) = 49.019, *p* = 0.000		

EI, exercise intention; EIF, exercise-induced feeling; EB, exercise behavior. ^**^p < 0.01.

## Discussion

Through correlation analysis and structural equation model analysis, this study found that exercise intention can positively predict exercise behavior, that is, the stronger the exercise intention, the higher the level of exercise behavior, which verifies Hypothesis 1. This result reaffirms the TBA’s view that intention to exercise is a valid predictor of exercise behavior, and it is consistent with the pre-epidemic research (Xiang, 2016; Liu et al., 2017). The reason is that the stronger the junior high school students’ exercise intention is, the higher the degree of effort they are willing to pay in the planned exercise behavior, and the more positive their attitude will be, which is bound to significantly improve the level of junior high school students’ exercise behavior. Therefore, in the post epidemic period, improving the exercise intention of junior high school students is still one of the main ways to improve exercise behavior.

### Moderating effect of openness personality

Bases on this result, this study also found that openness personality plays a regulatory role in the relationship between exercise intention and exercise behavior, which verifies Hypothesis 2. High level openness personality significantly enhances the predictive effect of exercise intention on exercise behavior, while low level openness personality weakens the predictive effect of exercise intention on exercise behavior, which once again proves that the executive power of different levels of openness personality is significantly different, consistent with previous studies (Pérez-Fuentes et al., 2019). The theory of personality traits points out that personality traits have the ability to guide behavior intention and individual behavior, and the behavior motivation that individuals choose or show when facing social behavior may directly originate from personality traits (Mao, 2019). The social communication ability and behavior motivation of the high level openness personality individuals are significantly higher than those of the low level openness personality individuals. In other words, individuals will be moderated by openness personality in the process of transforming from exercise intention to exercise behavior. The stronger the exercise intention of high-level openness personality individuals, the more motivated individuals will be and the more positive emotions they will experience, and the higher their ability to exercise behavior will be. Low level openness personality individuals, due to their low social interaction ability, lack of confidence, and dislike participating in social interaction activities (Chen and Liu, 2018), reduce the possibility of participating in exercise behavior, thus weakening the positive relationship between exercise intention and exercise behavior. This study suggests that, on the one hand, in the post epidemic period, we can stimulate junior high school students’ exercise intentions by enriching teaching content, actively organizing extracurricular sports activities and other diversified ways. On the other hand, we should strengthen the intervention of low level openness personality, promote its positive cognition and self-evaluation, improve its social interaction ability, and promote its active participation in physical exercise.

### Mediating effect of exercise-induced feeling

This study also found that the moderating of openness personality on the relationship between exercise intention and exercise behavior is realized through the mediator variable of exercise-induced feeling, which verifies Hypothesis 4. This result supports the theoretical view that feeling is the key factor to trigger and maintain the exercise intention of junior high school students, and is the key factor to affect exercise behavior (Brunet et al., 2018; Zhang and Chang, 2019).

The results show that after adding the mediator variable of exercise-induced feeling into the model, the moderating role of openness personality increases, and it can still significantly predict exercise behavior, which indicates that exercise-induced feeling plays a part of the mediator role in the relationship between openness personality and exercise behavior, which is consistent with previous research conclusions (Zhu and Dong, 2016). According to the theory of field dynamics, people are a field, and their psychological activities occur in a psychological field or living space. The direction and vector of individual behavior depend on the environmental stimulus and internal motivation of the individual. Individuals’ behavior is stimulated by internal motivation, exercise intention stimulates exercise behavior, and exercise-induced feeling and an openness personality stimulate individuals to be more willing to participate in exercise. The characteristics of openness personality, such as enthusiasm, good communication, activity, optimism and openness, can help junior high school students to encourage their ability to solve problems when encountering difficulties, give students full freedom of choice, and give students timely emotional interaction when they need emotional comfort. These supports meet the students’ development needs in three aspects of autonomy, competence and relationship, so as to enhance their exercise feeling. A high level of exercise-induced feeling can then predict a higher degree of exercise behavior. This result shows that exercise-induced feeling is an important mediator variable between exercise intention and exercise behavior. As an important variable at the level of individual non cognitive factors, it is not only affected by the external social environment, but also can effectively predict exercise behavior, playing a connecting role.

### Practical significance

The theoretical significance of this study is to expand the application of planned behavior theory in Chinese junior high school students. It explores the regulatory role of personality traits in the relationship between exercise intention and exercise behavior. Firstly, exercise intention remains an important predictor variable of exercise behavior, as well as exercise-induced feeling, which can mediate between exercise intention and behavior, while openness personality plays a moderating role in this process. It enriches the research related to the field of exercise intention and exercise behavior.

The practical significance of this study is to provide practical basis for Chinese junior high school students to improve their exercise behavior in the post epidemic period. Junior high school students are in the puberty stage. Students at this stage are more sensitive and vulnerable to interference from the external environment. The COVID-19 epidemic led to the closure of schools, which will cause a lot of damage to students. From the perspective of the theory of planned behavior, this paper discusses how personality traits and exercise induced emotions affect the transformation of junior high school students’ exercise intentions into exercise behaviors. Research shows that the open personality in personality traits can significantly enhance the transformation of exercise intentions into exercise behaviors. At the same time, exercise induced emotions play a mediating role in this process. This study also found that the exercise behavior of girls will be significantly lower than that of boys, which suggests that parents and teachers need to improve the exercise situation of girls and the reasons why girls do not like to exercise, so as to help them improve their exercise behavior and promote their physical and mental health.

## Limitations and prospectives

Firstly, the study on the mechanism of exercise intention on exercise behavior is based on cross-sectional study. Although cross-sectional research can effectively answer many types of questions and the findings can explain complex models, it is difficult to obtain causal relationships between variables in this type of study. In the future research, the longitudinal tracking design can be combined to be more effectively explain how exercise intention affects the exercise behavior of junior high school students. Secondly, the scope of this research is only Anhui Province, China. The research results may only apply to China or Asia, but not to the world. In future research, we can expand the sample range and conduct surveys around the world to obtain more authoritative results. Thirdly, this study focused primarily on junior high school students, and more samples are needed in the future, such as college students and adults. Despite these limitations, this study improves junior high school students’ exercise behavior by revealing the mediating and moderating mechanisms between exercise intention and exercise behavior.

## Conclusion

(1) In the post-epidemic period, intention to exercise significantly predicted exercise behavior in junior high school students, with the openness personality of junior high school students playing a moderating role in the process, i.e., influencing the strength and direction of the relationship. The stronger the intention to exercise, the lower the increase in exercise behavior for junior high school students with a low level of openness, and the stronger the intention to exercise, the higher the increase in exercise behavior for junior high school students with a high level of openness. It is important to be fully aware of the important role of openness in the process of exercise intention to promote exercise behavior.

(2) Exercise intention not only directly predicts exercise behavior, but also indirectly predicts exercise behavior through exercise-induced feeling, with openness personality playing a moderating role in this process. It suggests that in the post-epidemic era, not only the cultivation of exercise intention but also the cultivation of exercise-induced feeling in junior high school students should be emphasized in improving their exercise behavior.

## Data availability statement

The original contributions presented in the study are included in the article/supplementary material, further inquiries can be directed to the corresponding author.

## Ethics statement

This study was conducted in accordance with the Declaration of Helsinki, and it has been approved by the Human Research Ethics Committee of Huaibei Normal University.

## Author contributions

Q-SM collected and analyzed the data. Q-SM and S-JY wrote original draft preparation and revised the manuscript. H-RJ supported funding. All authors designed the study, contributed to the article, and approved the submitted version.

## Conflict of interest

The authors declare that the research was conducted in the absence of any commercial or financial relationships that could be construed as a potential conflict of interest.

## Publisher’s note

All claims expressed in this article are solely those of the authors and do not necessarily represent those of their affiliated organizations, or those of the publisher, the editors and the reviewers. Any product that may be evaluated in this article, or claim that may be made by its manufacturer, is not guaranteed or endorsed by the publisher.
